# *In vitro* biomechanical evaluation of a monocoque plate-spacer construct for cervical open-door laminoplasty

**DOI:** 10.1371/journal.pone.0204147

**Published:** 2018-10-18

**Authors:** Yukoh Ohara, Takeshi Hara, Alejandro A. Espinoza Orías, Satoshi Tani, Nozomu Inoue, Junichi Mizuno

**Affiliations:** 1 Center for minimally invasive spinal surgery, Shin-yurigaoka General Hospital, Kawasaki, Japan; 2 Department of Neurosurgery, Juntendo University School of Medicine, Tokyo, Japan; 3 Department of Orthopedic Surgery, Rush University Medical Center, Chicago, Illinois, United States of America; 4 Department of Neurosurgery, Jikei University School of Medicine, Tokyo, Japan; University of California Berkeley, UNITED STATES

## Abstract

**Purpose:**

To evaluate biomechanical differences between two surgical procedures for cervical open-door laminoplasty using human cadaveric spines.

**Methods:**

Twenty-four cervical vertebrae (C4-C6) from eight fresh-frozen human cervical spines were subjected to mechanical testing after being instrumented for open-door laminoplasty using a newly designed plate-spacer device with a monocoque structure (plate-spacer group; n = 12) or by conventional miniplate-alone fixation (miniplate group; n = 12). Cantilever bending testing was performed by applying a compressive load in the cranio-caudal direction to the base of the spinous process of the reconstructed laminar arch constructs until failure and strength and stiffness of the laminar arch were determined. The results are presented as mean ± standard deviation.

**Results:**

The plate-spacer group was approximately twice as strong as the miniplate group (27.6 ± 16.5 N vs. 13.5 ± 7.3 N, *p* < 0.05). Stiffness in the plate-spacer group exhibited the same trend (19.6 ± 9.3 N/mm vs. miniplate group: 11.4 ± 6.9 N/mm, *p* < 0.05).

**Conclusion:**

The fixation with the monocoque plate-spacer construct for open-door laminoplasty provided higher structural properties when compared against the plate-alone fixation. The spacer in the plate-spacer construct appears to contribute by preventing large deformations of the laminar arch caused by bending in cranio-caudal direction. Future studies will be required to investigate stress/strain distribution in the laminar arch constructs.

## Introduction

Multilevel cervical cord compression and myelopathy caused by degenerative diseases such as spondylosis and ossification of posterior longitudinal ligament is usually treated with posterior decompression surgical approaches. Cervical laminoplasty has gradually become a well-established surgical intervention since first introduction in 1983 [1]. Cervical laminoplasty was developed in response to the disadvantages presented by cervical laminectomy, including postoperative spinal instability causing kyphotic deformity and recurrent spinal canal compression by postoperative laminectomy membrane [1, 2]. In the original laminoplasty procedure outlined by Hirabayashi the lamina is reconstructed by using suture fixation. While long-term neurological results after cervical laminoplasty with suture fixation have been reported to be satisfactory [3, 4], lamina closure has been noted as a problem associated with this procedure. Matsumoto *et al*. reported that up to 34% of patients had some degree of closure of the lamina at one or more levels after laminoplasty using the suture fixation [5].

Spacers have been applied to laminoplasty to prevent lamina closure. Bone struts and ceramic and hydroxyapatite blocks have been used as a spacer element. However, dislodgement of the spacer without stable fixation is a concern, which could lead to subsequent premature laminoplasty closure and even root or cord compression if the spacer dislodges into the canal [6, 7].

As another approach to prevent laminar closure after the open-door procedures, plate fixation of the lamina has been introduced in laminoplasty. Frank *et al*. first described the use of miniplates adapted from cranial fixation systems to secure cervical laminoplasty [8]. The use of titanium miniplates to stabilize the posterior elements after laminoplasty was reported as a simple, durable, and effective technique to maintain the increased sagittal diameter of the spinal canal [9, 10]. Despite good overall clinical outcomes, the requirement of lengthy solid collar fixation [11] and high screw back-out rate after laminoplasty with plate-alone fixation have been reported in the literature [12].

Plate fixation has been used in conjunction with bone struts connected to the plate by screws, which provides immediate stabilization and prevents bone strut dislodgement. The bone struts are expected to recreate a continuous bony laminar arch spanning from one lateral mass to the contralateral one which cannot be achieved by laminoplasty using the plate alone. Goto *et al*. found that screw loosening was more common in cases without the spacers in the laminoplasty using plate fixation [13]. Shaffrey *et al*. reported on open-door laminoplasty fixed by titanium miniplate and allograft bone, emphasizing that the combination of titanium miniplates for immediate stabilization and allograft bone for long-term fusion led to no loss of canal decompression [14]. Despite improvements in reconstruction of the lamina by using this combination of spacer and plate fixation, this procedure is technically demanding and the longer than usual surgical time is of concern [15–19].

A new device for laminoplasty consisting of a titanium box-shaped spacer with two arms for plate fixation has been developed in response to the challenge of poor/unstable fixation by providing immediate stabilization and long term biological fixation by bony reconstruction of the laminar arch via bone induction of autogenous local bone chips inserted in the titanium spacer during surgery [20]. Clinical studies of the laminoplasty using this device showed good clinical outcomes, supported by radiological findings of bone formation around the spacer and hinge region [20–22]. The biomechanical benefits of this monocoque plate-spacer construct for stabilization in the laminoplasty have been investigated using finite element models [23]; however, to date, no biomechanical testing had been carried out in human cadaveric cervical spines.

The aims of the present study were to investigate biomechanical behavior of the laminar arch after cervical laminoplasty using the monocoque plate-spacer construct compared to the conventional miniplate alone as a standard using human cadaveric cervical spines.

## Methods

This cadaveric study was approved by IRB at Rush University Medical Center (ORA#13103105-IRB01). A total of 24 cervical vertebrae (C4-C6) were prepared from fresh frozen 8 human cervical spines obtained from a tissue bank (Lifelegacy Foundation, Tucson, AZ). The donor sample consisted of 4 men and 4 women with an average age of 56 ± 12 years (mean ± standard deviation). Computed tomographic (CT) scans were obtained to exclude samples that had anatomical abnormalities or pathological changes such as fractures, severe deformity, and metastatic disease. The specimens were wrapped in saline-soaked gauze and stored frozen at -20°C. The day before testing, the specimens were thawed overnight at room temperature.

**Fig 1:**
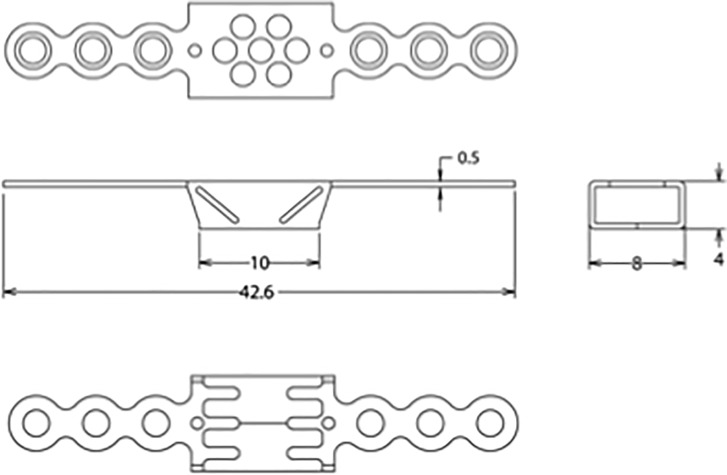
Drawing of a monocoque plate-spacer device consisting of two plates and a box-shape spacer. The spacer has small apertures allowing insertion of local autogenous bone chips and bone ingrowth for biological fixation. Reprinted from Neurol Med Chir (Tokyo). 2010;50(12):1132–6. under a CC BY license, with permission from Neurologia medico-chirurgia, original copyright 2010”.

**Fig 2:**
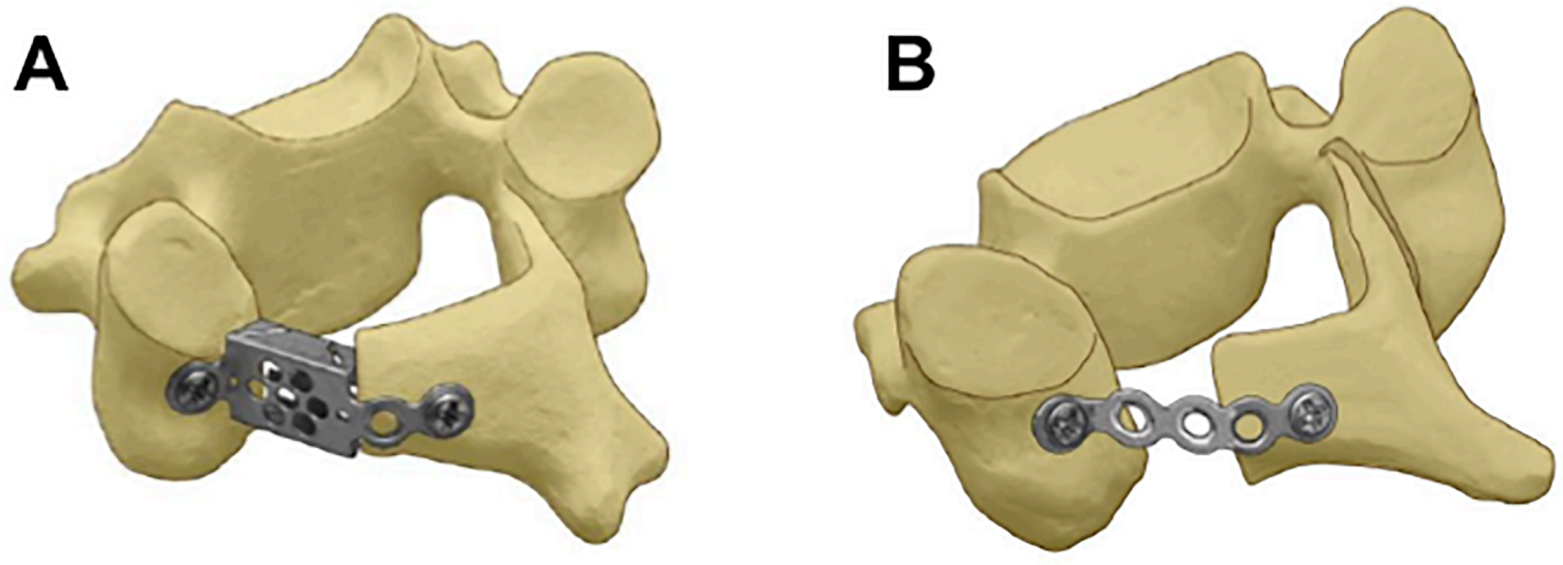
Schematic showing the device placement in laminoplasty. A: Monocoque plate-spacer device B: Miniplate.

The specimens were separated to individual vertebrae (i.e.; C4, C5 and C6) and laminoplasty surgery was performed for each vertebra by a single board-certified spine surgeon to keep consistency in the surgical procedure. A 3.0 mm wide gutter was made by high-speed cutting burr and a unicortical hinge was created. After creating a bicortical defect on the opposite side of the hinge, the laminar arch was reconstructed by two types of titanium-made devices: either a monocoque plate-spacer device (Laminoplasty Basket, Ammtec Inc., Japan, Figs 1 and 2) or a traditional miniplate device (Model no. 01–08220, Stryker Japan, Tokyo, Japan) (plate-spacer group; n = 12, miniplate group; n = 12). In both groups, the plates were bent so that the plates fit on the surface of the lateral mass and elevated lamina, forming the entire device in an open “Z” shape. A single screw (4 mm in length) was used for plate fixation at each end of the plate positioned at the lateral mass or elevated lamina for both groups. The vertebral body of each specimen was embedded in polymethylmethacrylate (Isocryl; Lang Dental, Wheeling, IL) for secure attachment to the testing frame, leaving the posterior elements free for mechanical testing.

**Fig 3:**
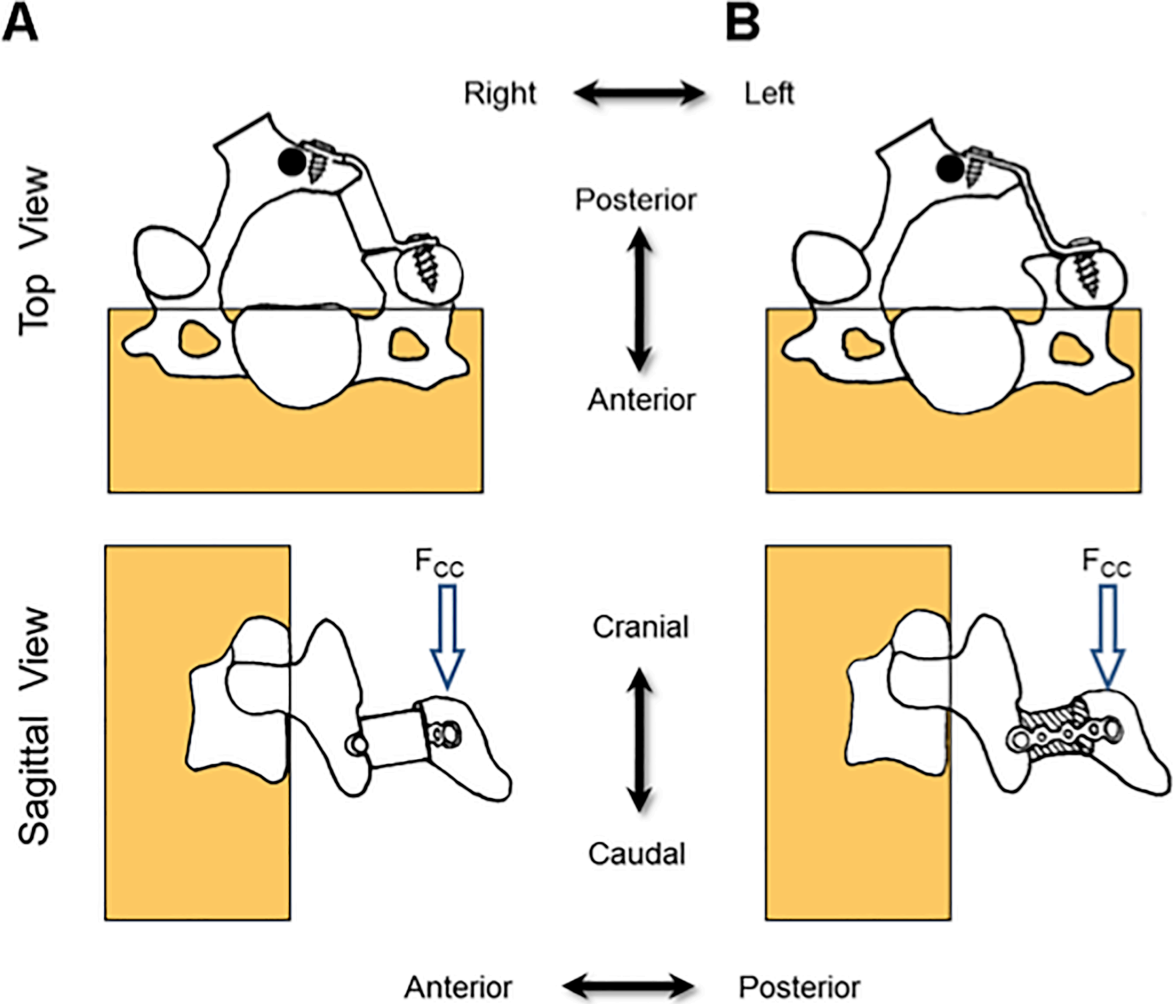
Schematic of the applied compressive loads: Top Row: Transverse plane view. Bottom Row: Sagittal view of both load configurations. Column A shows the plate-space device test, and column B is the miniplate device test. F_CC_ represents the force applied in the cranio-caudal direction. The loading point is shown as a black circle.

**Fig 4:**
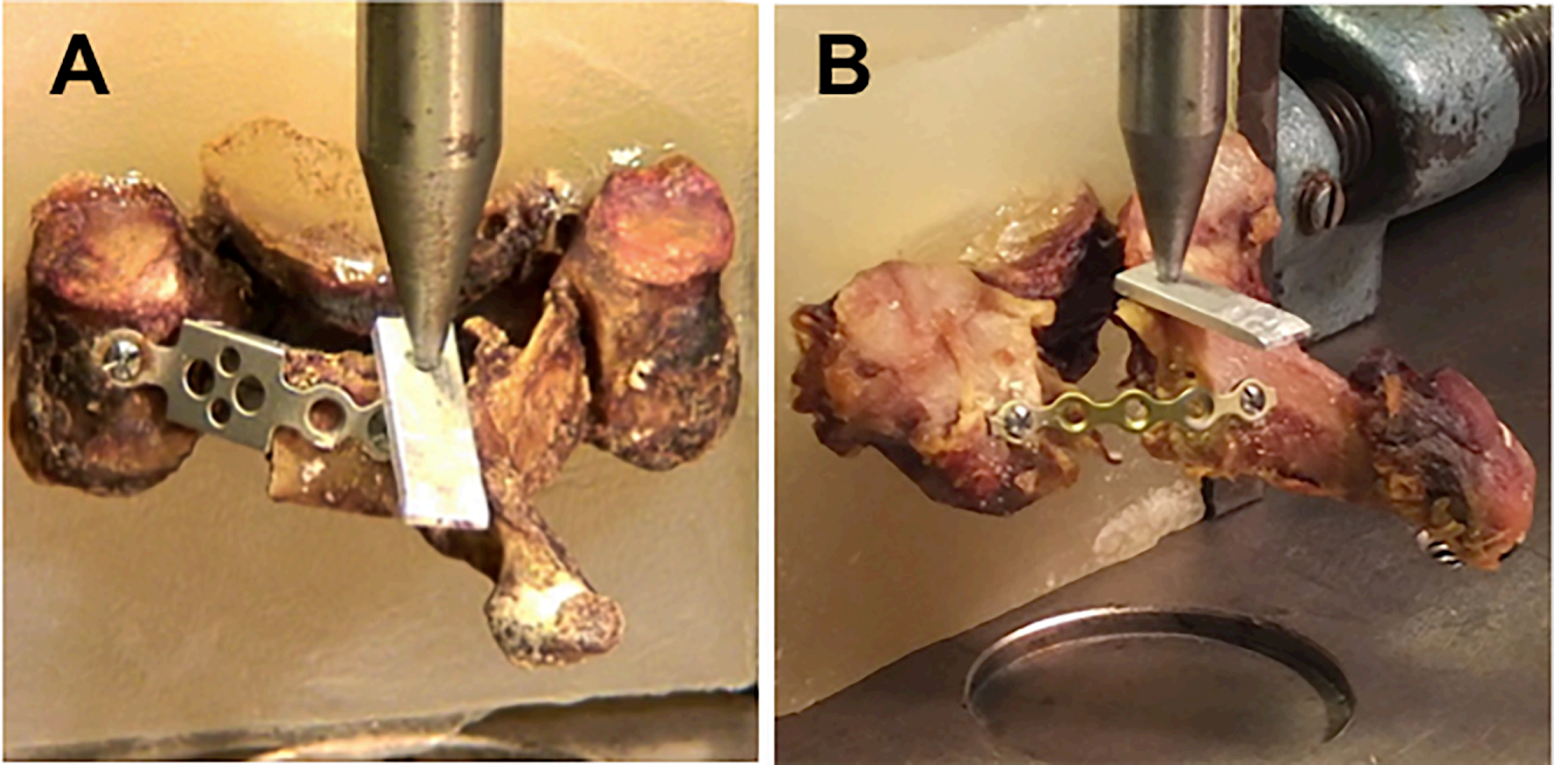
Experimental setup: An aluminum plate is placed between the lamina and an indenter with a semispherical tip to avoid penetration of the indenter into the lamina.

Cantilever bending testing was performed by applying compressive load to the center of the lamina in a cranio-caudal direction using a servohydraulic testing frame (Instron 8874; Instron Corp., Norwood, MA) running in displacement control with a cross-head speed of 1.0 mm/min until failure (Figs 3 and 4). A hemispherical indenter was used to apply compression loading through an aluminum plate with a hemispherical dimple, by which penetration of the indenter was prevented and large deformation of the lamina was allowed without constrain by the indenter (Fig 4).

The load to failure process was recorded by video during testing and macroscopic failure mode was evaluated. Load-displacement curves were recorded at 10 Hz. The fixation strength was determined by the initial peak of the load-displacement curve. Structural stiffness of the laminoplasty construct was determined by the slope of the initial linear portion of the load-displacement curve. Spinal level effects of the fixation strength and stiffness were studied by one way ANOVA with a Tukey’s post-hoc test. The fixation strength and stiffness were compared between the plate-spacer and the miniplate groups by an unpaired t-test. A significance level was set at *p* < 0.05. The results are presented as mean and standard deviation.

## Results

**Fig 5:**
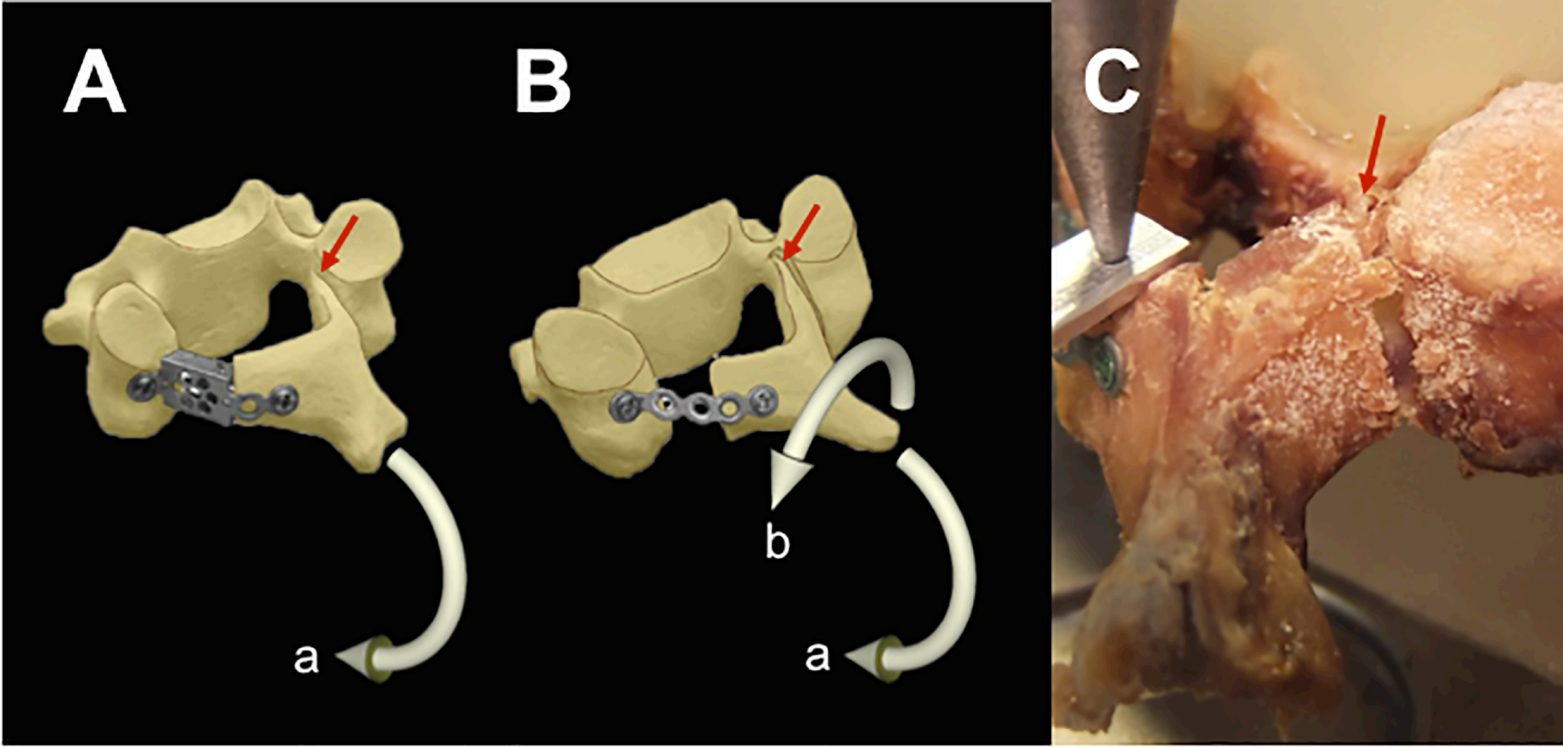
Failure mechanism of the laminoplasty reconstruct. Red arrows: Fracture initiation at the upper corner of the hinge. **A:** Monocoque plate-spacer device. The laminoplasty reconstruct rotated mainly in the sagittal plane (arrow **a**). **B:** Miniplate. In addition to the rotation in the sagittal plane (arrow **a**), rotation in the coronal plane (arrow **b**) is observed. **C:** Macroscopic observation of fracture initiation at the upper corner of the hinge in the miniplate sample.

No screw loosening was observed in any of the specimens. Failure was initiated at the upper corner of the hinge with an audible cracking sound in all specimens. In all miniplate group specimens, the miniplate was rotated as deflection of the elevated lamina increased during loading, which caused displacement of the cut-edge of the lamina towards the caudal direction. This phenomenon caused rotation of the lamina around the anteroposterior axis and, in turn, the hinge was twisted in the coronal plane in addition to the rotation in the sagittal plane. Conversely, these phenomena were only observed in a single specimen in the plate-spacer group, which showed gaps between the box-shaped spacer and the lateral mass or the cut-surface of the lamina. In the remaining specimens from the plate-spacer group, the spacer contacted to the lateral mass and cut-surface of the lamina during loading, which protected rotation of the lamina in the coronal plane (Fig 5).

**Fig 6:**
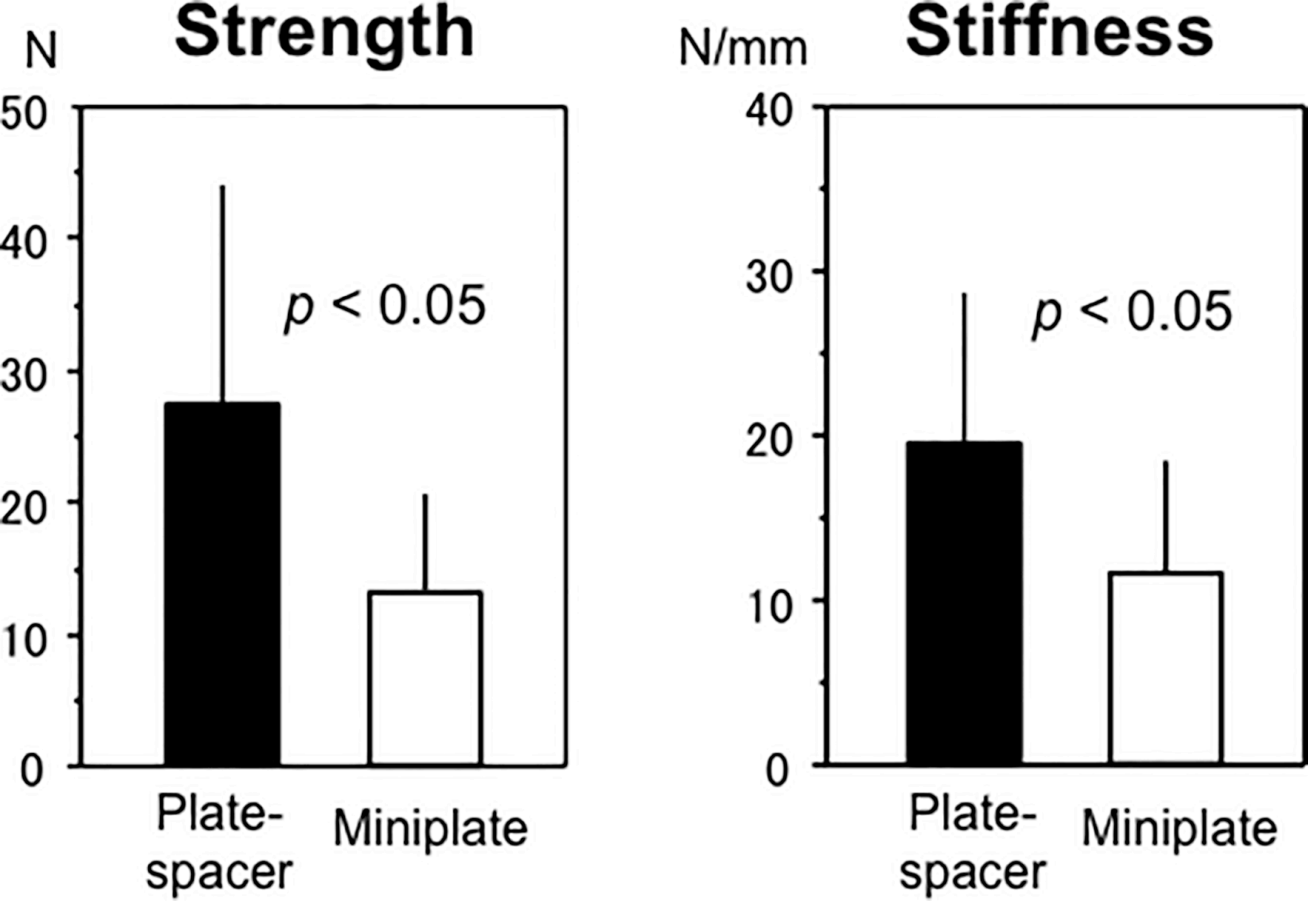
Results of mechanical testing. Error bar: standard deviation.

No statistical differences of the fixation strength and stiffness in each group were noted among the C4- C6 levels; thus, the following results were obtained from the pooled data from C4—C6. The plate-spacer group was approximately twice as strong as the miniplate group (27.6 ± 16.5 N vs. 13.5 ± 7.3 N, *p* < 0.05). The stiffness in the plate-spacer group exhibited the same trend (19.6 ± 9.3 N/mm vs. miniplate group: 11.4 ± 6.9 N/mm, *p* < 0.05) (Fig 6).

## Discussion

The present study demonstrated that the failure mechanism of the laminar arch reconstructed by either the plate-spacer or miniplate alone was hinge fracture under cantilever bending produced by compressive load in the cranio-caudal direction. However, the fixation strength and stiffness of the laminar arch with plate-spacer device were both approximately twice as high than those with the miniplate alone. Shear movement at the laminectomy gap under loading as presented by macroscopic deformation of the laminar cut-edge might have initiated hinge fracture starting at the gutter upper margin. In this study, single-screw fixation at the lateral mass and lamina ends was used in both the plate-spacer and miniplate groups based on clinical studies using one-screw fixation [11, 20]. However, several investigators recommend double-screw fixation at least in one side when plate-alone fixation is used [12, 18]. Furthermore, the screw length of 4 mm used in the present study was shorter than the screw length commonly used in the literature. The laminar arch reconstruction using the plate-alone fixation with double-screw fixation and longer screw length is thought to provide better structural strength and stiffness. Rotation of the plate about either of the screws’ axis is of concern when single-screw fixation is used. However, since the plate was bent in a shape of a wide-angle “Z” and therefore the axes of the screws did not coincide with the main axis of rotation of the plate, the effect of a weaker screw fixation with single and short screw length appears to be minimal. O’Brien reported no screw loosening was revealed radiographically at regular intervals during follow-up periods (1.5, 3, 6, 12 and 24 months) after laminoplasty using plate-alone and single-screw fixation in each side [9]. On the other hand, a high screw-back out rate of 16.4% (in 27 plates out of 27 plate fixation) was reported in double-screw fixation in laminoplasty using plate-alone fixation [12]. Similarly, a high rate of screw penetration, as high as 24.9%, to the cervical facet joint has also been reported in the laminoplasty using two-screw fixation [24]. Since the area for double-screw fixation on the lateral mass is limited and thickness of the lateral mass for the caudal screw is thinner, selection of the appropriate screw length is especially important for two-screw fixation on the lateral mass side [24]. Due to high complication related to double-screw fixation, the development of appropriate screw fixation technique including necessary number and length of screw is warranted as an important subject for future studies [24].

The higher strength and stiffness in the plate-spacer group appears to be brought by a more uniform load sharing provided by the box-like spacer, which is, effectively a stiffer structure than just the plate. A finite element analysis (FEA) was performed to investigate stress distribution and construct stability in cervical open-door laminoplasty models using the plate-spacer device used in the present study [23]. In this FEA study, a “plate-alone” model was also created by removing the box-shaped spacer except the base plate from the plate-spacer device to investigate effects of the spacer on the structural properties of the reconstructed laminar arch. The results of the stress analyses under postero-anterior or 66° laterally declined compressive loading conditions demonstrated that the box-shaped spacer contributed approximately 50% reduction of the stress at the hinge region by load transmission through bone-spacer interfaces at the elevated lamina and the lateral mass [25]. Although the loading conditions of the study are different from the present study, a similar mechanism by which the plate-spacer group provided higher strength and stiffness can be postulated, which is embodied by the increased structural stiffness of the box spacer. Additional finite element analysis using the same loading conditions as in the present study will be required to confirm this potential mechanism.

Biomechanical analyses of the laminoplasty construct under compressive loading in a postero-anterior direction have been reported in the literature [23, 25]. Maximizing construct strength under this loading condition is critical for the stability of the elevated lamina in order to prevent lamina closure [5, 26]. The stability of the reconstructed lamina in the cranio-caudal direction investigated in the present study is also important for preservation of extensor muscle function after laminoplasty. There are a growing number of investigators addressing roles of extensor muscles for prevention of axial neck pain and kyphotic deformity after laminoplasty [26–33]. When the extensor muscle attachments are preserved or reattached to the elevated laminae, tensile forces are applied in the cranio-caudal direction and the resulting bending moment is directly affects the laminar arch. In the present study, cantilever bending was produced by compressive loading in the cranio-caudal direction, which represents laminar arch bending by the extensor muscles. Cantilever bending causes the highest bending moment at the base of the cantilever and as such, this test protocol provides critical mechanical conditions for the gutter since it is located at the laminar arch. Clinical outcomes on the extensor muscles prevention procedure are controversial in the literature. Biomechanical studies on the laminar arch instability in the cranio-caudal direction may provide valuable information to analyze the mixed clinical outcomes of this procedure. The present study is the first to report, to the best of our knowledge, the biomechanical evaluation of the laminoplasty reconstruct in this critical loading condition.

The present study only used middle cervical levels of C4—C6 and the presented results did not include the level effect. A larger sample size may demonstrate the differences of the biomechanical parameters of the laminoplasty reconstruct. Since extensive multilevel laminoplasty including upper and/or lower cervical levels has been often performed recently, biomechanical studies including C2, C3 and C7 levels are also important. Future studies including C2—C7 levels with a sufficient sample size will be required to provide relevant information for extensive multilevel laminoplasty.

The biomechanical parameters studied in this study are limited to the overall structural characteristics and fracture mechanisms of the hinge and load transmission through the spacer were speculated by qualitative observation. Stress analysis of the hinge and the device-bone interfaces should be conducted to elucidate the failure mechanism of the laminar construct after laminoplasty. The present study used single vertebra experimental specimens to investigate individual vertebra fixation strength and stiffness of individual vertebra. However, multi-motion segments should be used to investigate structural properties of the cervical spine under different fixation techniques for multiple laminoplasty. Although the present study aimed to investigate initial fixation strength of the laminoplasty reconstruct after surgery, fatigue behavior of the construct should be investigated because early failure of the construct, such as screw loosening, could occur before biological fixation has been accomplished. The present study reported only the biomechanical evaluation of the open-door laminoplasty constructs using the newly-developed monocoque plate-spacer device and plate fixation techniques, even though a variety of other reconstruction techniques for laminoplasty is available. Nonetheless, the results of the present study would also be helpful to understand mechanical benefits of the other forms of plate-spacer constructs, i.e. bone graft strut or bone substitute such as hydroxyapatite and ceramic rigidly fixed to the plate, on the initial structural strength of the open-door laminoplasty constructs.

## Conclusion

Cervical open-door laminoplasty using a monocoque plate-spacer device provided approximately twice as much strength and stiffness as compared with plate-alone fixation under compressive loading applied to the base of the spinous process in cranio-caudal direction. Under this loading condition representing bending of the reconstructed laminar arch, all specimens failed from the upper corner of the hinge. While reconstructed laminar arch by plate-alone fixation exhibited coronal rotation under loading which caused twisting of the hinge, the plate-spacer device prevented this rotation by contact between the spacer and lamina or lateral mass during loading. Although further stress analysis will be required, load transmission through the spacer integrated in one structure appears to contribute to higher structural properties of the laminar arch after open-door laminoplasty.
